# Dynamin and reverse-mode sodium calcium exchanger blockade confers neuroprotection from diffuse axonal injury

**DOI:** 10.1038/s41419-019-1908-3

**Published:** 2019-09-27

**Authors:** Anton Omelchenko, Anil B. Shrirao, Atul K. Bhattiprolu, Jeffrey D. Zahn, Rene S. Schloss, Samantha Dickson, David F. Meaney, Nada N. Boustany, Martin L. Yarmush, Bonnie L. Firestein

**Affiliations:** 1 Department of Cell Biology and Neuroscience, Rutgers, The State University of New Jersey, 604 Allison Road, Piscataway, NJ 08854-8082 USA; 2 Neuroscience Graduate Program, Rutgers, The State University of New Jersey, 604 Allison Road, Piscataway, NJ 08854-8082 USA; 3 Department of Biomedical Engineering, Rutgers, The State University of New Jersey, 604 Allison Road, Piscataway, NJ 08854-8082 USA; 4 Department of Bioengineering, University of Pennsylvania, Philadelphia, PA 19104-6391 USA

**Keywords:** Assay systems, Apoptosis, Drug screening, Cell death in the nervous system, Trauma

## Abstract

Mild traumatic brain injury (mTBI) is a frequently overlooked public health concern that is difficult to diagnose and treat. Diffuse axonal injury (DAI) is a common mTBI neuropathology in which axonal shearing and stretching induces breakdown of the cytoskeleton, impaired axonal trafficking, axonal degeneration, and cognitive dysfunction. DAI is becoming recognized as a principal neuropathology of mTBI with supporting evidence from animal model, human pathology, and neuroimaging studies. As mitochondrial dysfunction and calcium overload are critical steps in secondary brain and axonal injury, we investigated changes in protein expression of potential targets following mTBI using an in vivo controlled cortical impact model. We show upregulated expression of sodium calcium exchanger1 (NCX1) in the hippocampus and cortex at distinct time points post-mTBI. Expression of dynamin-related protein1 (Drp1), a GTPase responsible for regulation of mitochondrial fission, also changes differently post-injury in the hippocampus and cortex. Using an in vitro model of DAI previously reported by our group, we tested whether pharmacological inhibition of NCX1 by SN-6 and of dynamin1, dynamin2, and Drp1 by dynasore mitigates secondary damage. Dynasore and SN-6 attenuate stretch injury-induced swelling of axonal varicosities and mitochondrial fragmentation. In addition, we show that dynasore, but not SN-6, protects against H_2_O_2_-induced damage in an organotypic oxidative stress model. As there is currently no standard treatment to mitigate cell damage induced by mTBI and DAI, this work highlights two potential therapeutic targets for treatment of DAI in multiple models of mTBI and DAI.

## Introduction

Traumatic brain injury (TBI) is a worldwide leading cause of death and disability^1^, with concussion, or mild TBI (mTBI), the most common^2,3^. Although TBI refers to abnormal brain function or neuropathology due to physical impact, it is comprised of multiple interrelated, pathological processes, resulting in diverse symptoms and morbidities. Primary injury, or damage directly from impact, may be focal or diffuse^4^. Inertial forces from rotational head motions lead to shearing and stretching of axons and induce diffuse axonal injury (DAI), a common TBI pathology^5^. In DAI, breakdown of the axonal cytoskeleton results in the formation of axonal swellings and bulbs, impeding recovery due to disruption of protein and organelle trafficking. While DAI has been long recognized as a significant pathology in moderate and severe TBI^5–7^, it is now considered the principal neuropathology of mTBI with evidence from animal model^8^, human pathology^9^, and non-invasive human neuroimaging studies^10–13^.

**Fig. 1:**
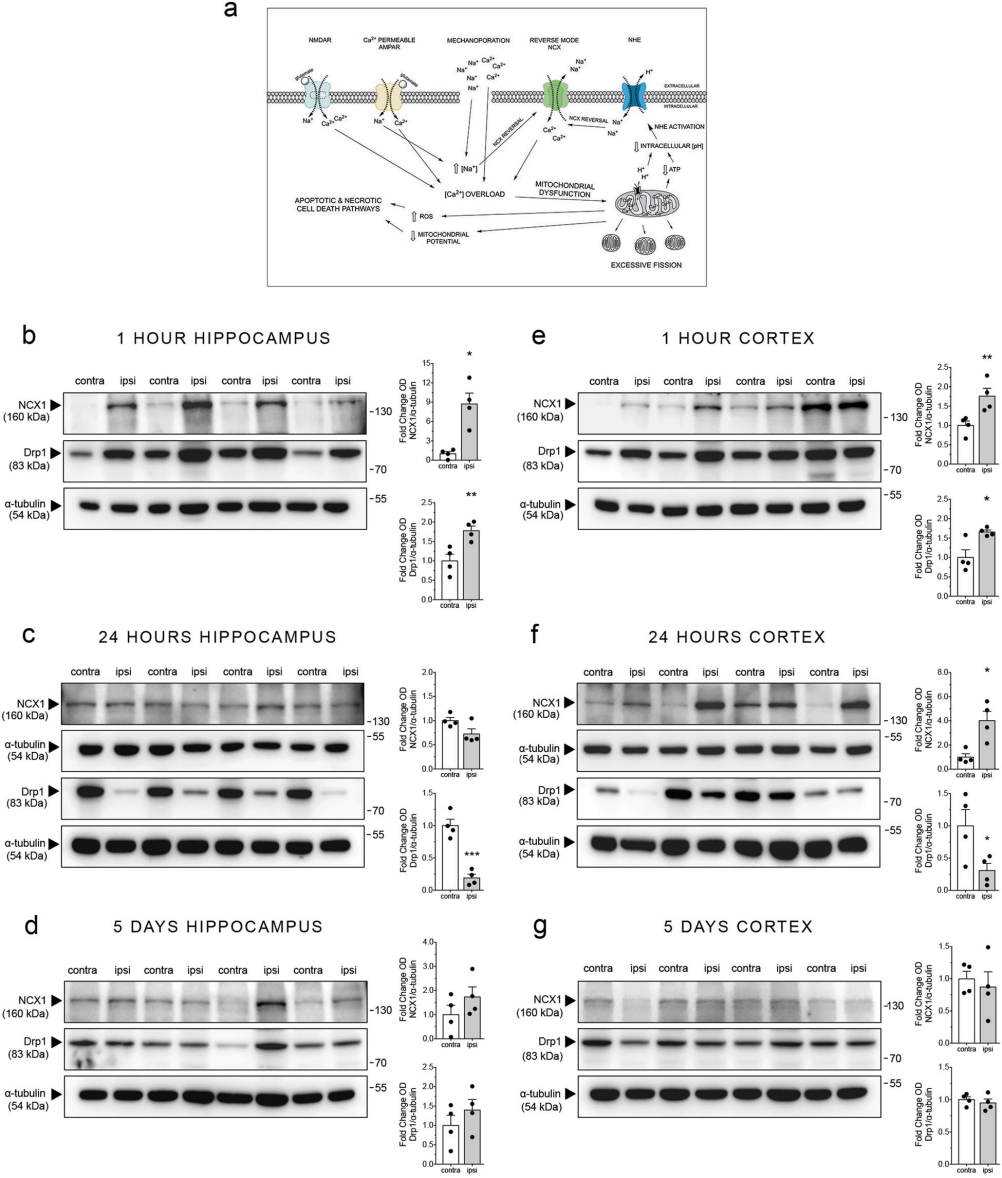
NCX1 and Drp1 protein expression are differentially upregulated and downregulated in the ipsilateral hippocampus and cortex at different time points post-mTBI in vivo. **a** Schematic of the hypothesized secondary-phase TBI molecular cascade. Excess glutamate release following TBI induces aberrant neuronal depolarization and acidosis. Acidosis induces activation of the sodium hydrogen exchanger (NHE), which combined with ionotropic glutamate receptor activity and mechanoporation leads to dysregulation of intracellular Na^+^ balance. The resulting increase in intracellular Na^+^ activates the reverse-mode of NCX in neurons, which contributes significantly to excessive influx of Ca^2+^. Excessive Ca^2+^ influx through NCX, *N*-methyl-d-aspartate (NMDA) glutamate receptors, and Ca^2+^-permeable α-amino-3-hydroxy-5-methyl-4-isoxazolepropionic acid (AMPA) glutamate receptors leads to aberrant Ca^2+^ signaling, propagating mitochondrial dysfunction. Elevated intracellular Ca^2+^ is sequestered by mitochondria and leads to aberrant mitochondrial fission, increased reactive-oxidative species production, and a decrease in mitochondrial potential and ATP production. These cascades ultimately induce apoptotic and necrotic cell death. **b**–**g** Mice were subjected to mTBI using a modified controlled cortical impact technique that models the mechanics of mTBI and were euthanized 1 h, 24 h, or 5 days post-injury. Brains were quickly removed and dissected. Tissue from the ipsilateral (with respect to injury) and contralateral hippocampus and cortex was isolated and flash frozen. Protein expression was analyzed by western blot analysis. **b** Representative western blots and respective densitometric quantification showing NCX1 and Drp1 protein expression in the ipsilateral and contralateral hippocampus 1 h post-mTBI. **c** Western blots and respective quantification showing NCX1 and Drp1 protein expression in the hippocampus 24 h after mTBI. **d** Western blots and quantification showing NCX1 and Drp1 protein expression in the hippocampus 5 days following mTBI. **e**–**g** Representative western blots and respective densitometric quantification showing NCX1 and Drp1 protein expression in the cortex **e** 1 h post-mTBI, **f** 24 h post-mTBI, and **g** 5 days post-mTBI. Error bars are s.e.m. *n* = 4 mice per group, *p < 0.05, **p < 0.01, by paired Student’s *t* test

Disruptions in sodium (Na^+^) and calcium (Ca^2+^) ion homeostasis contribute to neuropathology of TBI and DAI^5,14^. Excessive glutamate release and ionotropic glutamate receptor activation, membrane mechanoporation, and acidosis-mediated overactivation of Na^+^/H^+^ exchangers (NCX) induce a rise in intracellular Na^+^^15,16^. Under basal conditions, NCX regulate ionic balance via expulsion of one Ca^2+^ in exchange for three Na^+^ ions in forward mode. During conditions of excess intracellular Na^+^, NCX reverse mode is activated, resulting in Ca^2+^ influx^15,17^. Accumulation of intracellular Ca^2+^ and dysregulated Ca^2+^ homeostasis are critical events in secondary axonal injury^18,19^. Aberrant activation of *N*-methyl-d-aspartate receptors increases intracellular Ca^[2+16,19^ propagating mitochondrial dysfunction; generation of reactive oxygen species (ROS); and activation of calpain and other proteases, phospholipases, and endonucleases^20^, leading to degradation of cytoskeletal and axonal membrane proteins and, ultimately, axonal degeneration (Fig. 1a)^21^.

Mitochondrial dysfunction is a critical component of TBI, mTBI, and DAI biopathology^19,22^. Mitochondria regulate Ca^2+^ homeostasis by sequestering intracellular Ca^2+^, and excessive mitochondrial Ca^2+^ adsorption hinders ATP production, induces oxidative stress, and leads to apoptosis^23–26^. TBI induces impairments in mitochondrial respiration in the ipsilateral hemisphere by 1 h and up to 14+ days post-injury^23,27^. Human mitochondria at the center of the injury exhibit degenerative changes in structural morphology, characterized by mitochondrial budding and formation of spherical mitochondrial fragments ≤24 h post-TBI^28^. Mitochondrial aggregation occurs inside dendritic and axonal swellings away from injury^28^. By 3 days post-TBI, most mitochondria within the injury site exhibit end-stage degenerative morphology, while mitochondria within the periphery are reactive and degenerate^28^. In addition, mitochondria in the ipsilateral cortex and hippocampus undergo differential time-dependent changes post-TBI^29^. Damage to cortical mitochondrial propagates faster, such that a significant proportion is eliminated in the cortex, but not in the hippocampus, by 24 h post-TBI^29^.

Since mitochondrial dysfunction is a key component of mTBI and DAI pathology, we analyzed protein expression after controlled cortical impact (CCI) in an in vivo animal model of mTBI to investigate changes in potential targets involved in mTBI and DAI pathogenesis. We found time-dependent differential expression of NCX1, the ubiquitously expressed sodium–calcium exchanger, and dynamin-related protein1 (Drp1), a GTPase responsible for mitochondrial fission, in ipsilateral cortex and hippocampus post-mTBI. As DAI is a hallmark component of mTBI, we tested pharmacological agents that block NCX1 and Drp1 using a microfabricated culture platform of in vitro traumatic axonal injury^30,31^. Our data show that dynamin inhibitor dynasore and reverse NCX blocker SN-6 attenuate stretch-induced axonal varicosities and mitigate mitochondrial fragmentation and rounding. Using an in vitro slice model of H_2_O_2_-induced cell death, we found that only dynasore confers neuroprotection. Taken together, our work is the first to demonstrate neuroprotection via dynasore and SN-6 after axonal strain injury. Our work highlights two potential therapeutic targets for treatment of DAI.

## Results

### Expression of NCX1 and Drp1 protein changes following mTBI in vivo

As deleterious alterations in mitochondrial morphology and function are key components of mTBI pathology (Fig. 1a), we investigated changes in Drp1 and NCX1 expression post-CCI. There are conflicting reports on how Drp1 expression changes post-TBI^32,33^. Furthermore, there are no studies addressing NCX1 expression post-TBI. Our analysis shows upregulation of NCX1 and Drp1 protein in hippocampus at 1 h, but not at 24 h, post-mTBI (Fig. 1b). In contrast, Drp1 expression decreased 24 h post-mTBI (Fig. 1c). NCX1 and Drp1 expression returned to control levels 5 days post-mTBI (Fig. 1d). Cortical NCX1 and Drp1 expression increased 1 h post-mTBI (Fig. 1e). NCX1 expression increased and Drp1 expression decreased 24 h post-mTBI (Fig. 1f). NCX1 and Drp1 expression returned to control levels at 5 days post-mTBI (Fig. 1g). Taken together, these data suggest similar delay in mitochondrial pathology in the cortex and hippocampus following mTBI, as reported for traditional TBI^29^.

### Dynamin and NCX blockade mitigates secondary axonal injury in an in vitro model of DAI

**Fig. 2:**
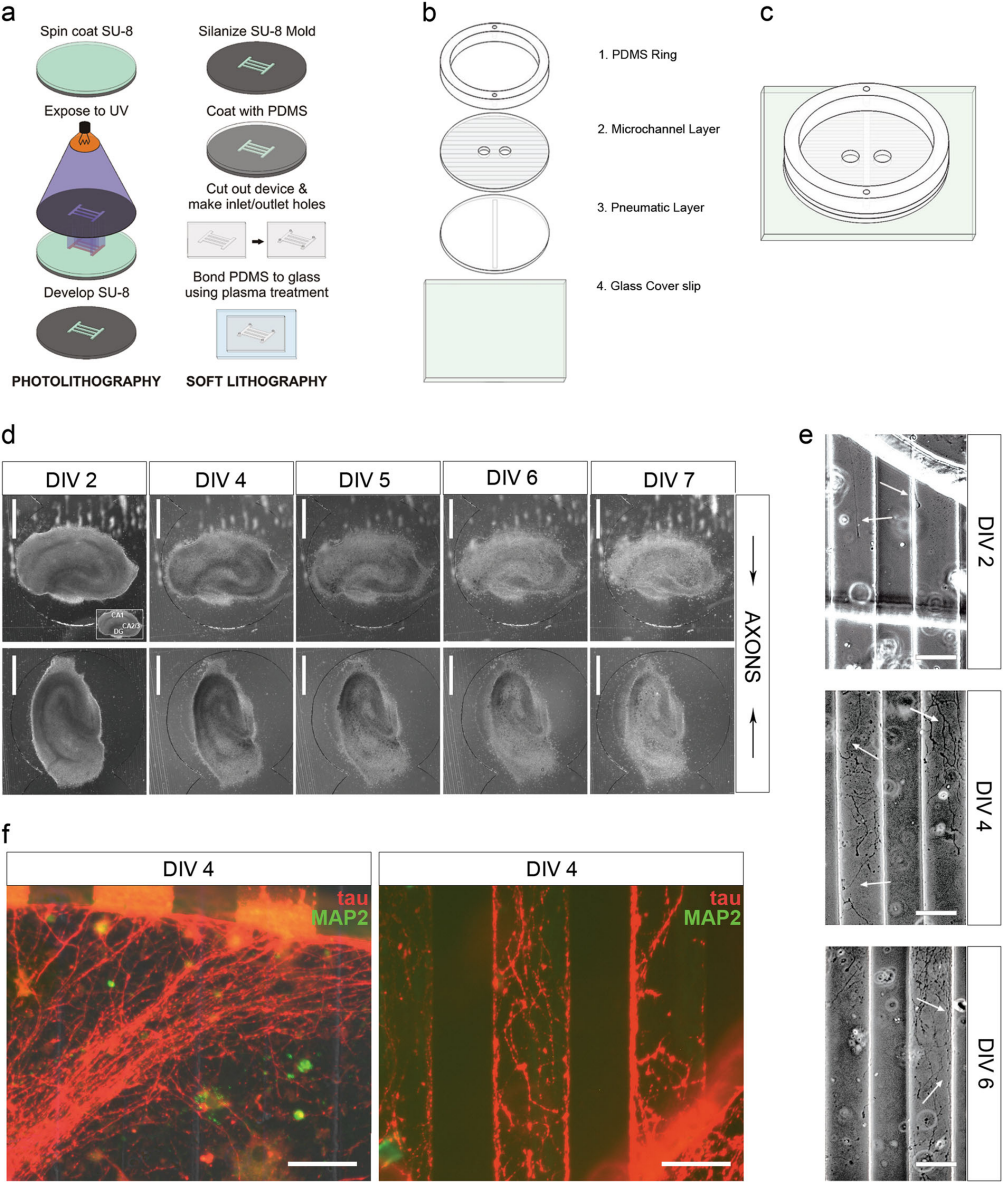
Microfluidic culture device for high-throughput screening of pharmacological agents to facilitate development of DAI therapeutics. **a** Diagram of microfabrication process. **b** Schematic of the four layer components that comprise the microfluidic culture device. **c** Schematic of assembled microfluidic device. **d** Representative phase-contrast images of organotypic hippocampal slice cultures grown in the microfluidics device. Each individual device hosts two organotypic slice cultures, and each slice faces the interconnecting microchannels with the dentate gyrus or CA3 region of the hippocampus, respectively. **e** Phase-contrast images of axonal projections growing through the interconnecting microchannels between the two culture compartments at various days in vitro. **f** Fluorescence micrographs of immunostaining against tau protein and MAP2. Processes entering and inside of microchannels are immunopositive for tau and negative for microtubule-associated protein 2 (MAP2). Scale bars, 500 μm (**d**), 50 μm (**e**), 50 μm (**f**)

As DAI is a principal mTBI component, we investigated the effects of axonal stretch on Drp1 activity and NCX1 localization in an in vitro DAI model. We developed a microfabricated organotypic culture device to model axonal strain injury for high-throughput screening of potential DAI therapeutics^30,31^, which we re-designed for more straightforward assembly (Fig. 2). We used hard and soft photolithography (Fig. 2a) to produce three polydimethylsiloxane (PDMS) layers, which are assembled and permanently bonded to coverglass (Fig. 2b, c). Organotypic hippocampal slices are cultured within separate compartments with the dentate gyrus of one slice and CA3 of the other facing interconnecting microchannels (Fig. 2d). Axons extend into adjacent microchannels and travel across to the proximal well containing the other slice (Fig. 2e). Processes entering microchannels express tau (Fig. 2f), axonal marker, and are negative for microtubule-associated protein 2, dendritic marker (Fig. 2f).

**Fig. 3:**
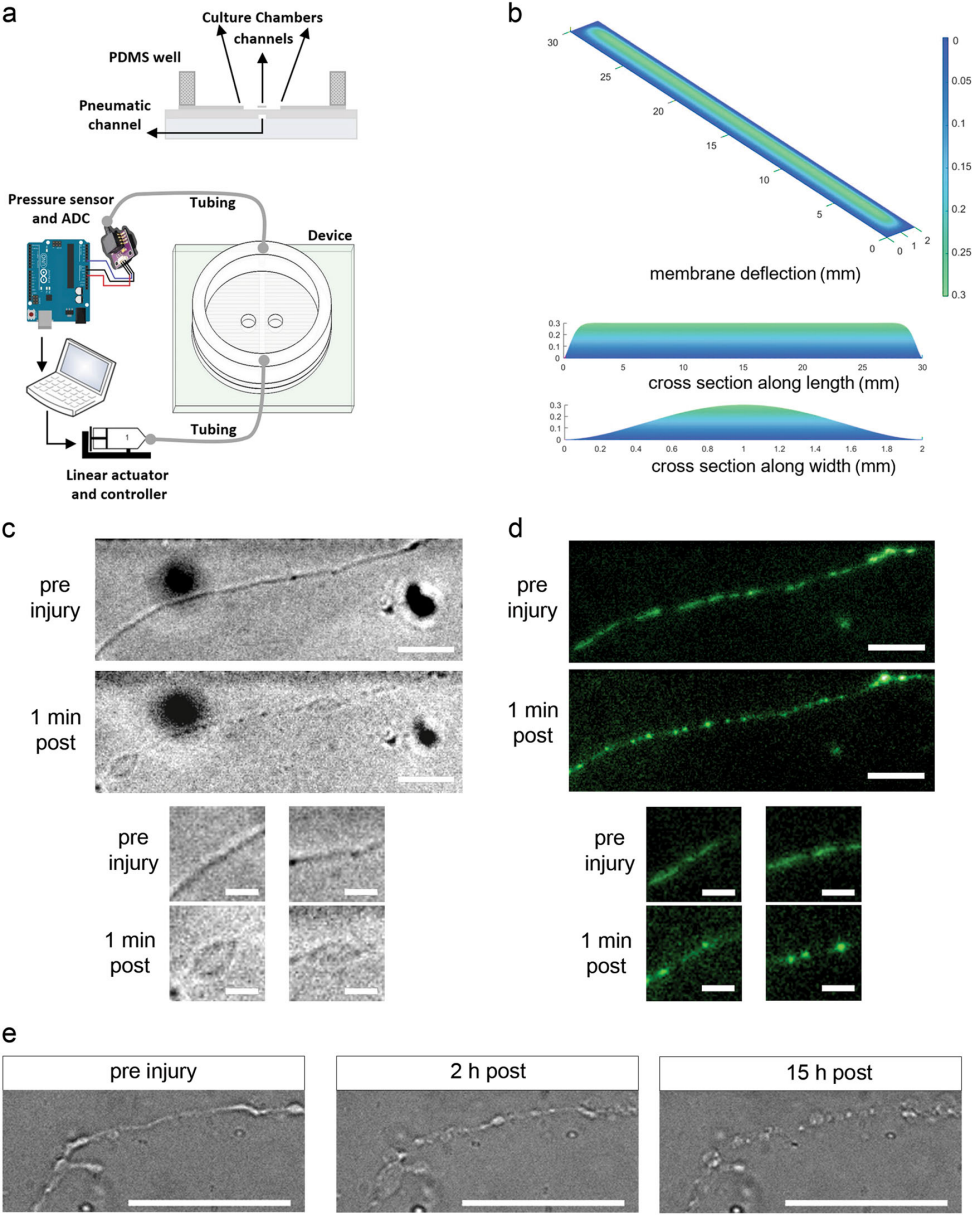
Application of 15% strain induces formation of axonal varicosities and mitochondrial fragmentation. **a** Schematic displaying the application of uniaxial strain injury. A linear actuator drives a syringe piston to apply pneumatic actuation and inject pressure into the cavity beneath the interconnecting microchannels. Injected pressure is read using a high-speed analog to digital converter. **b** Strain modeling in MATLAB, PDE Toolbox. Application of 2.0 psi induces a deflection of 300 μm in 2-mm-wide pneumatic channel (15% strain). **c** Phase-contrast micrographs of processes before and ~1 min after application of 15% strain injury. **d** Fluorescence micrographs of mitochondria (labeled with MitoTracker Green FM) within interconnecting microchannels before and after application of 15% strain injury. **e** Phase-contrast micrographs of axonal processes before and 2 and 15 h after application of 15% strain injury. Scale bars, 25 μm, 10 μm (**c**), 25 μm, 10 μm (**d**), 50 μm (**e**)

Axonal strain injury is applied by injecting pressure into the pneumatic cavity beneath the axonal microchannel via syringe piston driven by linear actuator controlled with the custom software (Fig. 3a). We use finite element analysis to determine strain produced by deflection of the PDMS membrane from applied pressure^30,31^. Here the thickness of each pneumatic and microchannel layer is 79 ± 3 µm, and we calculated strain produced by deflection of the PDMS membranes with combined thickness of the two layers when 2.0 psi pressure is applied. This pressure creates a vertical membrane deflection of 299.9 µm (Fig. 3b). Peak membrane deflection occurs in 22 ms, resulting in 15% strain with a rate of 6.81 s^−1^. We reported that 10%, 25%, and 45% strain induces axonal varicosities, a hallmark neuropathology of clinical DAI^8,30,34^. Here we chose 15% strain to screen potential DAI therapeutics as this rate does not produce transection and consistently induces formation of axonal beads and subsequent degeneration, hallmarks of traumatic axonal injury. To investigate the effects of axonal strain injury on mitochondrial dynamics, we labeled mitochondria with Mitotracker Green, applied 15% strain injury, and assessed axonal and mitochondrial morphology using phase-contrast and fluorescence microscopy, respectively. Application of 15% strain injury led to the formation of axonal bulbs and beads at 1 min post-injury (Fig. 3c). In addition, mitochondrial fragmentation and rounding occurred at 1 min following injury (Fig. 3d), similar to fragmentation reported from glutamate-induced excitotoxicity^35^. As axonal bulbs and beads swell and increase in size and ultimately disintegrate following injury (Fig. 3e), our data suggest that mitochondrial dysfunction correlates with secondary injury phase of DAI.

To investigate the effects of axonal stretch injury on Drp1, we applied 15% strain injury and fixed cultures 5 min post-injury. We observed increased Drp1 and TOMM20 (mitochondrial marker) colocalization within injured axons post-stretch injury (Supplementary Fig. S1). Since translocation of Drp1 to outer mitochondrial membrane is the first step in Drp1-dependent mitochondrial fission, these results suggest that Drp1-mediated fission plays a role in secondary axonal injury^36^. We next examined NCX1 localization post-stretch injury. NCX1 was clustered along axonal processes in control cultures; however, post-stretch injury, NCX1 localized to axonal beads (Supplementary Fig. S2). These results motivated us to pharmacologically target Drp1 and NCX1 activity to improve axonal viability post-stretch injury.

**Fig. 4:**
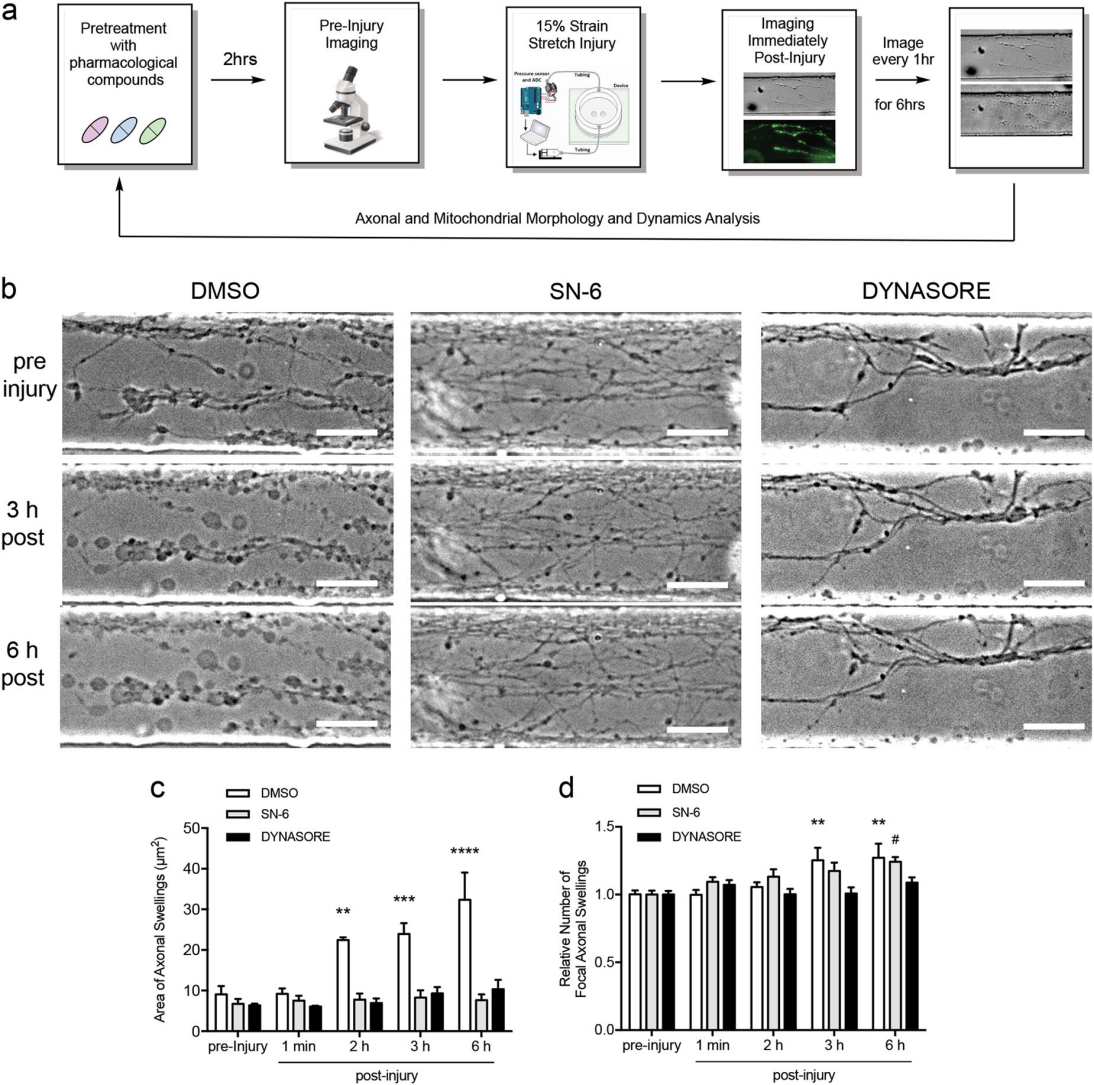
SN-6 and dynasore mitigate increased swelling of axonal varicosities following axonal strain injury. **a** Experimental design. **b** Representative phase-contrast micrographs of axonal pathology following injury (15% strain) and vehicle (0.08% DMSO) or drug pretreatment. **c** Application of axonal stretch to control (vehicle treated) cultures induces the formation of large (twofold) focal axonal varicosities by 2 and 3 h post-injury. Axonal swellings continue to increase in size (threefold) through 6 h post-injury. Pretreatment with 80 μM dynasore or 10 μM SN-6 prior to stretch prevents injury-induced swelling of axonal varicosities at 3 and 6 h. **d** Strain injury induces a substantial increase in the number of varicosities at 3 and 6 h post-stretch in control cultures. Pretreatment with 80 μM dynasore prevents the increase in number of varicosities at 3 and 6 h post-injury. Pretreatment with SN-6 delays the increase in varicosity number until 6 h post-injury. Asterisk (*) and hash (#) indicate significant difference from respective condition’s pre-injury data. Results obtained from three independent experiments, (*n* = 3). ^#^*p* < 0.05, **p < 0.01, ***p < 0.001, ****p < 0.0001 by two-way ANOVA followed by Bonferroni post-hoc test, 30–40 regions of interest (ROIs) were randomly chosen from three independent microfluidic device cultures (separate litter donor animals) per condition. Error bars are s.e.m. Scale bar, 50 μm

To determine whether inhibition of Drp1 or NCX1 mitigates axonal swelling post-injury, we treated slices within devices with dynasore, inhibitor of dynamin1, dynamin2, and Drp1 GTPase activity^26^, and SN-6, selective NCX inhibitor^37,38^, or vehicle, performed pre-injury phase-contrast (axonal morphology) and fluorescence (labeled mitochondria) microscopy, applied 15% strain injury, and imaged immediately post-injury and every hour for 6 h (Fig. 4a). Treatment was performed prior to injury to ensure compounds diffuse into microchannels containing axons. Medium containing compounds remained in the device throughout experiments. Dynasore or SN-6 prevented injury-induced increases in bead size at 6 h post-injury (Fig. 4b, c). In contrast, only dynasore treatment prevented an increase in the number of swellings while cultures treated with SN-6 developed an increase in the number of swellings by 6 h post-injury (Fig. 4d), similar to that seen in control cultures at 3 and 6 h. These results suggest that reverse-mode NCX and dynamin or Drp1 play distinct roles in the molecular cascade following axonal strain injury and contribute to the formation of axonal bulbs and beads.

### Dynasore and SN-6 prevent mitochondrial fragmentation immediately post-injury

**Fig. 5:**
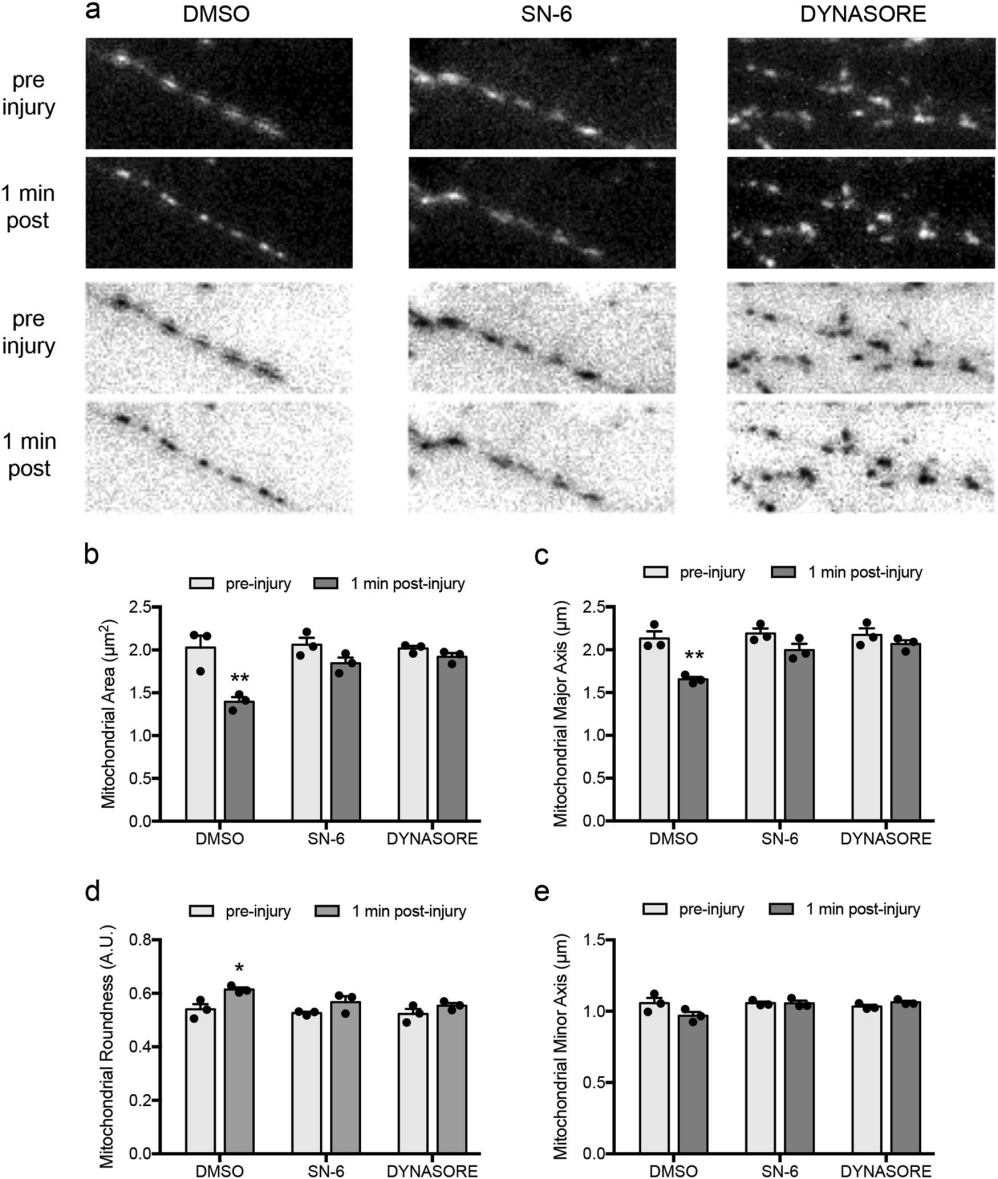
SN-6 and dynasore prevent fragmentation and rounding of axonal mitochondria post-strain injury. **a** Representative fluorescence micrographs of Mitotracker Green FM-labeled axonal mitochondria, per respective condition, before and 1 min after stretch injury. **b**–**e** Pretreatment with dynasore (80 µM) or SN-6 (10 µM), but not vehicle, prevents injury-induced reduction in mitochondrial area and mitochondrial major axis and increase in mitochondrial roundness. Results obtained from three independent experiments, (*n* = 3), 100–150 ROIs per condition), *p < 0.05, **p < 0.01, by two-way ANOVA followed by Tukey’s multiple comparisons test. Error bars are s.e.m. Scale bars, 5 μm

We reported that application of axonal strain leads to a significant rise in mitochondrial membrane potential hours post-injury^30^. This, in tandem with mitochondrial fragmentation we observed, led us to examine the effects of dynasore and SN-6 on mitochondrial dynamics post-injury. We compared morphology of axonal mitochondria before and 1 min post-injury respective to drug treatments. Treatment with dynasore or SN-6 prevented mitochondrial fragmentation immediately post-injury (Fig. 5a–d). In control cultures, application of 15% strain injury resulted in decreased mitochondrial area (Fig. 5b) and length of mitochondrial major axis (Fig. 5c), suggesting mitochondrial fragmentation. Both dynasore and SN-6 treatment prevented these decreases (Fig. 5a, b). Application of 15% strain injury also resulted in increased mitochondrial roundness, and treatment with dynasore or SN-6 prevented this change (Fig. 5d). Mitochondrial minor axis was unaffected by injury (Fig. 5e). Taken together, these data implicate reverse-mode NCX1 and dynamin or Drp1 GTPase activity in the molecular cascade leading to mitochondrial fragmentation post-injury.

### Dynasore, but not SN-6, attenuates oxidative stress-induced cell death

**Fig. 6:**
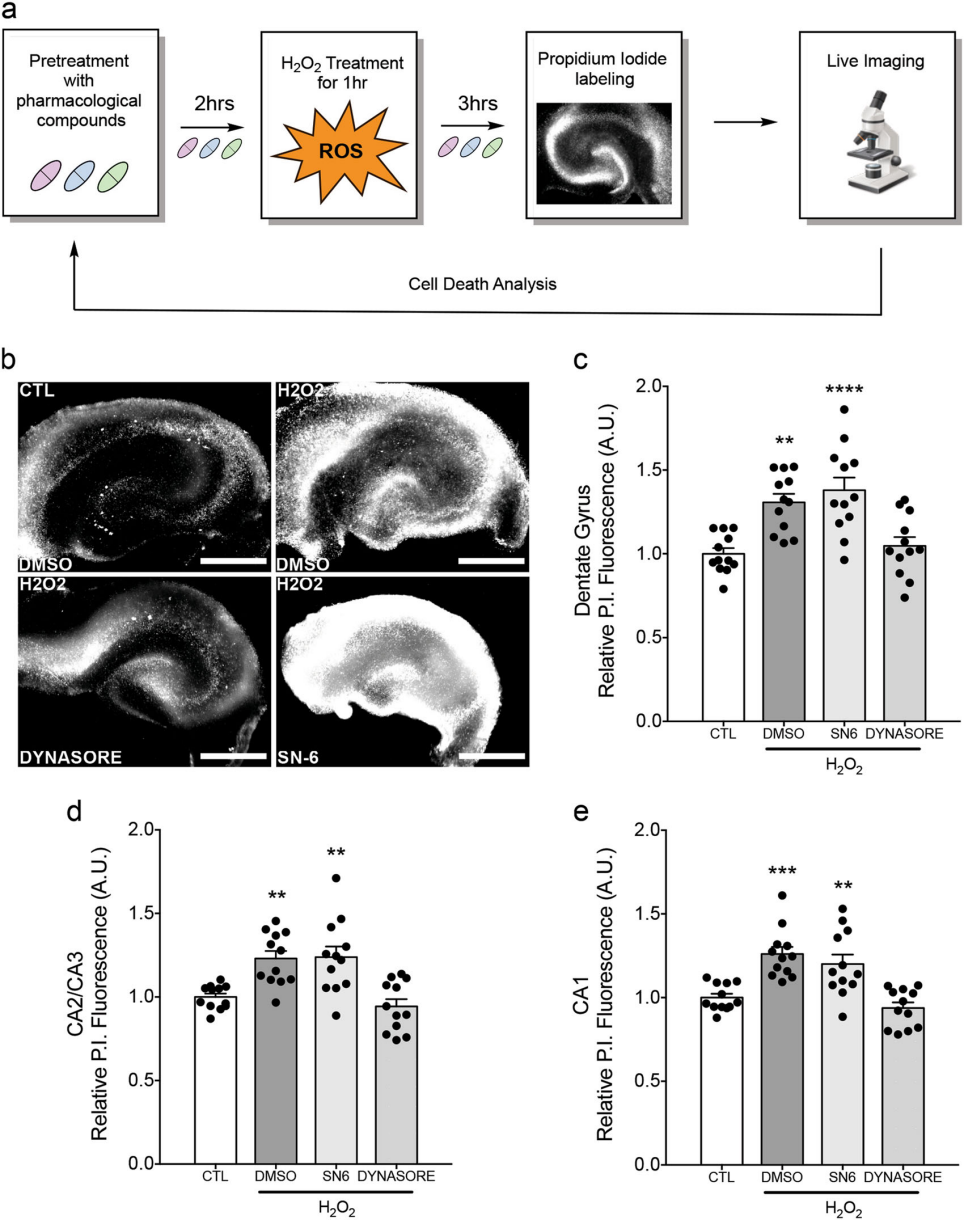
Dynasore attenuates oxidative stress-induced cell death. **a** Experimental design. Organotypic hippocampal slice cultures (DIV 2–3) were pretreated with pharmacological compounds or 0.08% DMSO and cell death was assessed live 3 h after exposure to H_2_O_2_ via relative propidium iodide (PI) fluorescence. **b** Representative images of P.I.-labeled cell death in respective organotypic hippocampal cultures following H_2_O_2_ exposure. **c** Dynasore (80 µM), but not SN-6 (10 µM), treatment significantly attenuates H_2_O_2_-induced cell death in the dentate gyrus, **d** CA2/CA3, and **e** CA1 regions. Results obtained from three independent experiments, *n* = 3. Each experiment comprised of 4 organotypic slices per condition (each from separate donor animal), 48 slices total with 12 per condition. **p < 0.01, ***p < 0.001, ****p < 0.0001 by one-way ANOVA followed by Bonferroni post-hoc test. Error bars are s.e.m. Scale bars, 500 μm

As our data show that dynasore and SN-6 alter the outcome of axonal injury, we next examined whether they provide protection from oxidative stress-induced cell death, a model of downstream secondary injury cascade, using an organotypic H_2_O_2_ injury model. We used the same paradigm as axonal stretch experiments; however, hippocampal slices were grown in culture plates, treated with 2.5 mM H_2_O_2_ for 1 h, and stained with propidium iodide (PI) to measure cell death (Fig. 6a). As with previous experiments, drug-containing medium was present throughout the experiment. H_2_O_2_-induced oxidative stress led to significant cell death in all hippocampal regions (Fig. 6b). As hypothesized, dynasore treatment attenuated H_2_O_2_-induced cell death in all hippocampal regions (Fig. 6c–e). However, SN-6 treatment did not ameliorate H_2_O_2_-induced cell death in any region. These data support a model where reverse-mode NCX function plays a role upstream of oxidative stress in the secondary injury cascade, and inhibition of dynamin and Drp1 GTPase activity is protective against oxidative stress.

### Dynasore attenuates rise in ROS post-H_2_O_2_ exposure

**Fig. 7:**
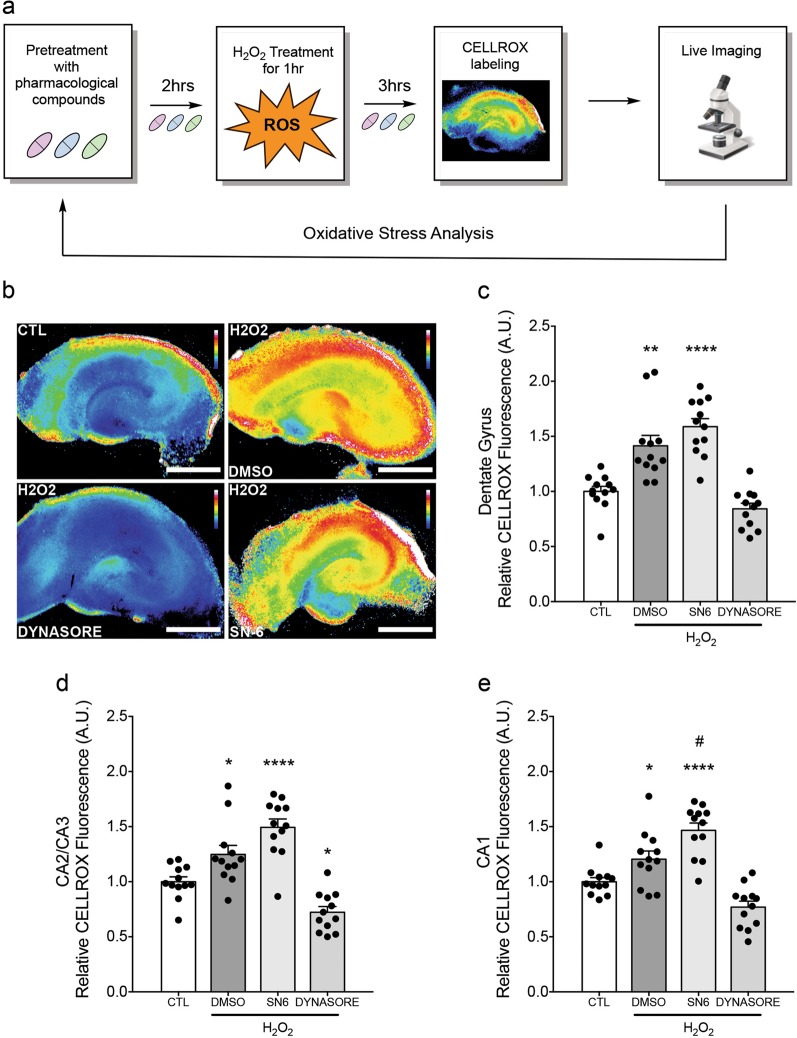
Dynasore treatment mitigates rise in ROS following H_2_O_2_ exposure. **a** Experimental design. Organotypic slice cultures (DIV 2–3) were pretreated with pharmacological compounds or vehicle and relative oxidative stress was assessed live 3 h after H_2_O_2_ exposure using CellROX™ Green Reagent. **b** Representative pseudocolored (16-color LUT in ImageJ) micrographs of CellROX™ Green fluorescence in respective cultures following H_2_O_2_ exposure. **c** Dynasore (80 µM), but not SN-6 (10 µM), treatment significantly attenuates H_2_O_2_-induced oxidative stress in the dentate gyrus, **d** CA2/CA3, and **e** CA1 regions. Asterisk (*) and hash (#) indicate significant difference from controls and DMSO groups, respectively. Results obtained from three independent experiments, *n* = 3. Each experiment comprised of 4 organotypic slices per condition (each from separate donor animal), 48 slices total with 12 per condition. *p < 0.05, ^#^*p* < 0.05, **p < 0.01, ****p < 0.0001 by one-way ANOVA followed by Bonferroni post-hoc test. Error bars are s.e.m. Scale bars, 500 μm

Scavengers of free radicals block mitochondrial permeability transition and apoptosis, suggesting that mitochondrial ROS generation and subsequent mitochondrial permeability transition play crucial roles in apoptosis^26^. To investigate whether neuroprotection by dynasore attenuates oxidative stress, staining with fluorescent CellROX post-H_2_O_2_-induced injury was used to measure relative ROS (Fig. 7a). Consistent with cell death results, dynasore, but not SN-6, attenuated ROS in all hippocampal areas (Fig. 7b–e). Furthermore, dynasore decreased oxidative stress to below control condition in CA2/CA3 (Fig. 7b, c) while SN-6 increased oxidative stress to levels above vehicle in CA1 (Fig. 7b, e). These data suggest that inhibition of dynamin and Drp1 GTPase activity, but not reverse-mode NCX, attenuates injury-mediated ROS production and further support the idea that attenuation of cell death by dynasore is mediated through attenuation of ROS production.

### SN-6 and dynasore induce distinct changes to pro- and anti-apoptotic protein expression

Drp1-mediated fission plays an important role in apoptosis and precedes caspase activation and cytochrome C release^39,40^. Moreover, caspase-mediated cleavage of NCX and other membrane calcium pumps occurs during apoptosis and necrosis^41,42^. To investigate whether dynasore or SN-6 treatment affects the expression of proteins responsible for apoptotic signaling, we performed western blot analysis of lysates from hippocampal slices exposed to H_2_O_2_ and either dynasore or SN-6. Tissue was isolated and lysed 3 h following H_2_O_2_ exposure, and the same drug paradigm was used as for cell death and oxidative stress studies.

**Fig. 8:**
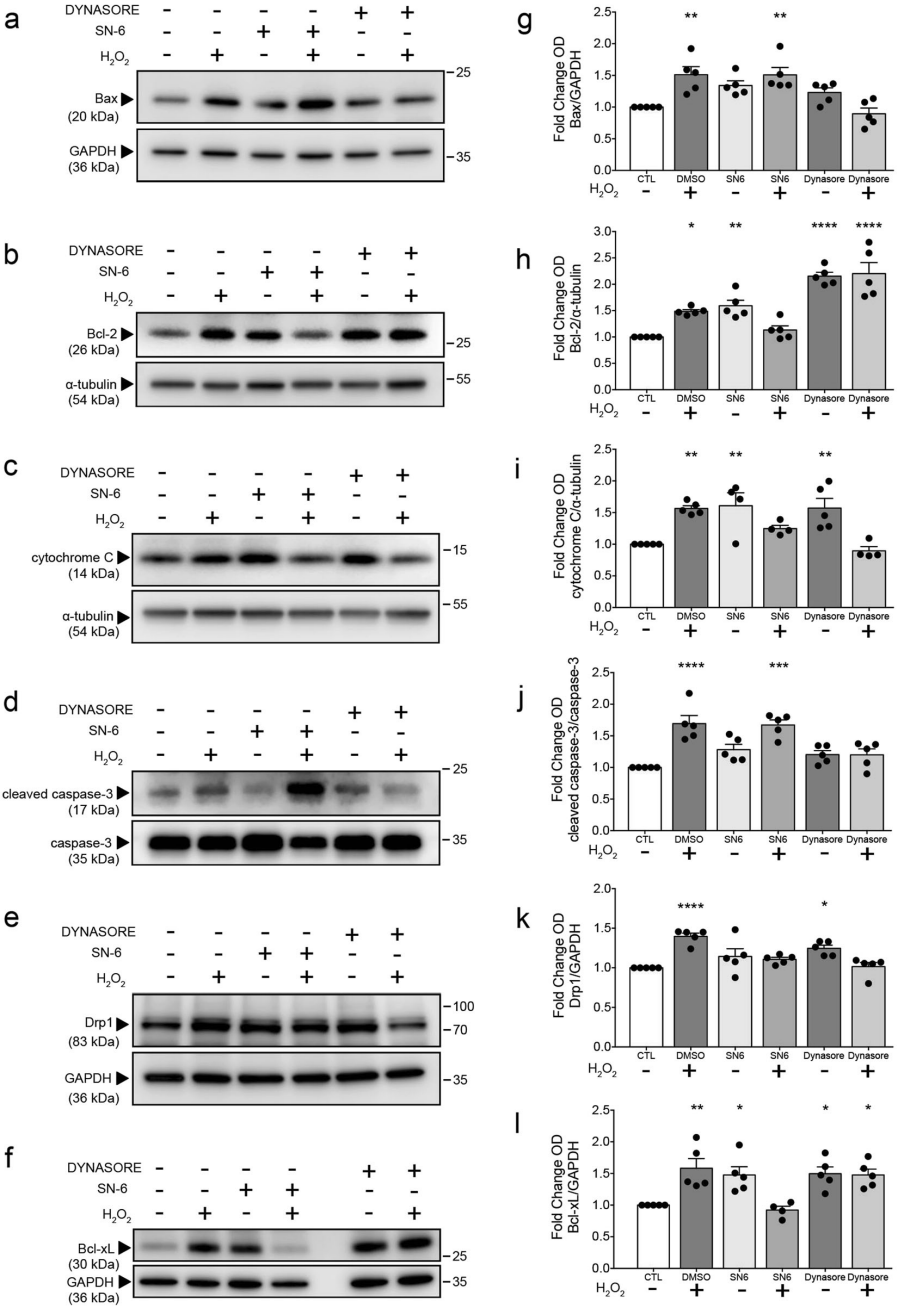
SN-6 and dynasore treatment differently affects the expression of pro- and anti-apoptotic proteins under baseline and oxidative stress conditions. The same model and experimental paradigm described above and western blot was used to assess shifts in protein expression in response to drug and H_2_O_2_. Co-cultures of four adjacent organotypic hippocampal slices (DIV 2–4) obtained from the same donor animal (P4–P8 rat) were used to prepare each tissue lysate sample. **a**–**f** Representative western blots of hippocampal slice lysates showing the expression of **a** Bax, **b** Bcl-2, **c** cytochrome C, **d** cleaved p17 caspase-3, **e** Drp1, and **f** Bcl-xL. **g**–**l** Densitometric quantification of the respective western blot analysis. GAPDH and α-tubulin were used as internal loading controls. Cleaved-caspase expression was compared to total caspase-3. Data were normalized to respective control from each independent experiment. Data were obtained per five independent experiments (different litters across experiments), *n* = 5. *p < 0.05, **p < 0.01, ***p < 0.001, ****p < 0.0001 by one-way ANOVA followed by Tukey’s multiple comparisons test. Error bars are s.e.m.

H_2_O_2_-induced oxidative stress significantly increased Bax expression, and treatment with dynasore, but not SN-6, attenuated this increase (Fig. 8a, g). Treatment with dynasore or SN-6 under control conditions upregulated Bcl-2 expression, while only dynasore maintained increased Bcl-2 expression following H_2_O_2_-induced oxidative stress (Fig. 8b, h). Interestingly, while exposure to H_2_O_2_ or treatment with dynasore or SN-6 alone upregulated cytochrome C expression, treatment with dynasore or SN-6 attenuated H_2_O_2_-induced increases in cytochrome C expression (Fig. 8c, i). H_2_O_2_ exposure increased cleaved caspase-3 (p17) expression, and dynasore, but not SN-6, mitigated this increase (Fig. 8d, j). Furthermore, H_2_O_2_ exposure significantly increased Drp1 expression, and dynasore or SN-6 diminished this effect (Fig. 8e, k). Surprisingly, dynasore treatment alone led to a modest increase in Drp1 expression, and this effect was not observed when dynasore treatment occurred with H_2_O_2_ exposure. Bcl-2 and Bcl-xL expression was augmented by H_2_O_2_ exposure, and H_2_O_2_-promoted increases were diminished by SN-6 (Fig. 8f, l). However, like Bcl-2, treatment with dynasore or SN-6 alone upregulated Bcl-xL expression to levels comparable to H_2_O_2_-induced levels. It is important to note that treatment with SN-6 alone upregulated the expression of both Bcl-2 and Bcl-xL only under baseline conditions, and this effect was diminished under oxidative stress. Taken together, these data suggest that neuroprotective effects of dynasore are mediated through alterations to pro- and anti-apoptotic signaling protein expression. Although we hypothesize that the protective effects of SN-6 are primarily due to decreased Ca^2+^ influx resulting from inhibition of reverse-mode NCX early in secondary axonal injury, it is surprising that SN-6 treatment alone under baseline conditions induced Bcl-2 and Bcl-xL expression and that SN-6 treatment under oxidative stress attenuated H_2_O_2_-induced upregulation of Drp1.

## Discussion

TBI is a leading cause of death and disability, and there are no available therapeutics to mitigate secondary cell damage outside impact site and prevent subsequent neurological dysfunction. Here we report two pharmacological compounds as potential mTBI and DAI therapeutics. Our findings suggest a key role for mitochondrial fragmentation in DAI and highlight mitigation of Ca^2+^ overload and mitochondrial dysfunction as two promising strategies for TBI and mTBI.

Analysis of NCX1 and Drp1 proteins suggests delay in mitochondrial-related pathology between cortex and hippocampus post-mTBI. In context, upregulation of cortical Drp1 post-TBI was reported in a weight-drop mouse TBI model, at 1 h, peak expression at 24 h, and decreasing, yet still upregulated, expression through 7 days post-TBI^32^. However, a different study reported decreased Drp1 expression in whole-brain ipsilateral hemisphere at 6, 24, 48, and 120 h post-mTBI and increased Drp1 expression at the same time points post-severe TBI^33^. Our data complement these results, and while weight-drop TBI is not mild, our results are consistent, suggesting rapid changes to cortical and hippocampal mitochondrial function following mTBI. mTBI induces transient debilitation of mitochondrial function, recovering between 48 h and 5 days post-injury, while severe TBI causes mitochondrial dysfunction characterized by continuous energy deficit and hyperglycolysis^33,43^. During transient period of mitochondrial dysfunction post-mTBI, a subset of mitochondria does not operate at maximum capacity until sufficient fusion restores baseline function^33,43^. However, these studies included 6 h as the earliest time point post-injury and examined Drp1 expression in the entire injured hemisphere; we analyzed tissue within 1 mm from injury site. Our data suggest graded mitochondrial dysfunction post-mTBI with respect to distance from injury site. Mitochondrial fission is excessive proximal to injury during immediate hours post-injury, but these processes are recoverable and return to normal function days post-injury^44,45^.

Our work builds upon a previous study using another stretch injury model showing induction of robust rise in intra-axonal Ca^2+^ from the extracellular milieu and that treatment with bepridil, a non-selective calcium channel blocker, which also inhibits NCX, moderately reduces Ca^2+^ entry post-stretch^14^. Taken together, these data support a therapeutic effect of SN-6 due to decreased Ca^2+^ influx, resulting from selective inhibition of aberrantly activated reverse-mode NCX post-stretch injury.

Increased extracellular ROS leads to axoplasmic Ca^2+^ overload and formation of axonal spheroids on sites containing aggregates of reverse NCX1, N-type Ca^2+^ channels, and actin^46^. Others have reported therapeutic benefit of SN-6 and selective inhibitors of NCX reversal mode in in vivo TBI models. NCX blockade prior to TBI in rodents prevents astrocyte loss in CA2/CA3 hippocampal regions^47^. Our study is the first to show axo-protection by SN-6 post-stretch injury and further highlights reverse-mode NCX as a molecular target for DAI and mTBI therapies.

In parallel to study of NCX1, several groups investigated Drp1 inhibition as potential treatment for DAI. Using a weight-drop model, it was reported that intraperitoneal injection of a selective Drp1 inhibitor (mdivi-1) at 10 min post-injury prevented TBI-induced behavioral deficits, cell death, and edema^32^. Mdivi-1 also mitigated TBI-induced changes to mitochondrial morphology, reduced cytoplasmic translocation of cytochrome C, and decreased caspase-3 activation^32^. However, a recent study reported that mdivi-1 does not affect Drp1 GTPase activity or mitochondrial elongation^48^ and, instead, inhibits mitochondrial complex I respiration and blocks generation of ROS by reverse electron transfer^48^. In addition to non-selective dynamin inhibitors, such as dynasore, the sole alternative to mdivi1 is the selective peptide inhibitor P110, which blocks Drp1 GTPase activity and interaction of Drp1 with partners. Few studies have investigated P110 and dynasore as therapies for TBI. While treatment with P110 provides neuroprotection by preventing aberrant mitochondrial fission and ROS generation, there are several issues with in vivo applications of P110 as it is a peptide^49^. P110 inhibits mitochondrial fission only at high doses (1 mM) in vitro, compared to mdivi1 (25 μM)^50^. Moreover, P110 administration in vivo was not effective in an animal model of pulmonary arterial hypertension^50^. As peptide therapeutics have short plasma half-life and low oral bioavailability, optimization of in vivo delivery and stability of P110 are needed.

Literature regarding neuroprotection by dynamin inhibitors, such as dynasore, is limited. Augmented recovery of motor function by immediate intraperitoneal injection of dynasore following spinal cord injury (SCI) was reported in vivo^51^. Dynasore prevents SCI-induced neuronal apoptosis by inhibiting pro-apoptotic cell death pathways^51^. As dynasore is a non-selective dynamin inhibitor and inhibits dynamin1 and dynamin2 in addition to Drp1, it is possible that the effects of dynasore may be due to changes to clathrin-coated endocytosis, cholesterol homeostasis, and Golgi vesiculation^52^. Dynasore has dynamin-independent effects, such as disrupting lipid rafts and destabilizing F-actin^53,54^. As it has been previously shown that lipid raft alterations by cholesterol depletion promotes neuronal survival and axonal regeneration following trauma^55^, it is possible that the dynamin-independent effects of dynasore may mediate neuroprotection via these other mechanisms. Further investigation of the selectivity of, and possible neuroprotection by, dynamin and Drp1 inhibitors is necessary.

Careful consideration should be taken when designing mTBI therapies based on inhibition of Drp1 and NCX1 function. Transient increased expression of Drp1 and NCX1 at 1 h post-mTBI followed by decreased Drp1 expression and continued elevated NCX1 expression 24 h after trauma and subsequent return of both proteins to normal levels at 5 days post-mTBI suggests that therapeutics targeting Drp1 and NCX1 function would need to be administered shortly after trauma. As a subset of mitochondria does not function at maximum capacity post-mTBI until baseline function is reestablished^33,43^, therapeutics targeting aberrant mitochondrial fission should be administered during this early critical window. Moreover, during the mTBI neurometabolic cascade, intracellular Ca^2+^ levels spike significantly shortly post-mTBI and remain elevated until 3–4 days post-trauma^56^. Taken together with our findings showing elevated cortical NCX1 expression 24 h post-mTBI, therapeutics, such as NCX1 inhibitors, that attenuate elevated Ca^2+^ may demonstrate a longer therapeutic window than those that target excessive mitochondrial fission. These considerations are critical as incorrectly timed administration may be detrimental for restoration of baseline neurophysiology and function.

Our data showing attenuation of cell death and oxidative stress by dynasore post-H_2_O_2_ exposure suggest that neuroprotection provided by dynasore may stem from inhibition of Drp1-mediated aberrant mitochondrial fission and excessive ROS production. Our crude lysate western blot analysis reveals prevention of H_2_O_2_-induced increased Bax and cleaved-caspase-3 expression by dynasore and prevention of increased Drp1 expression by SN-6. These effects may contribute to improved viability and decreased ROS following H_2_O_2_-mediated insult observed here, as other studies have made similar conclusions based on comparable data^51,57–59^. It is important to note that SN-6 and dynasore treatment increased cytochrome C, Bcl-2, and Bcl-xL expression under baseline conditions. Dynasore treatment alone also increased Drp1 expression, likely due to compensatory upregulation as a result of Drp1 activity inhibition. While SN-6 inhibits plasma membrane NCX1 and not mitochondrial NCX^60^, it is possible that inhibition of reverse-mode NCX1 under baseline conditions affects Ca^2+^ homeostasis and thus the expression of mitochondrial proteins. In addition, SN-6 inhibits NCX2 and NCX3, which show differential expression in glia and neurons^61^ and contribute significantly to Ca^2+^ regulation in the brain. Inhibition of NCX isoforms under baseline conditions may affect synaptic function and neuron–glia interactions^61–63^. The effects of these drugs under baseline conditions must be better understood before considering possible translational work.

Taken together, data presented here provide additional evidence for the importance of Ca^2+^ overload and mitochondrial dysfunction in the molecular underpinnings of DAI and highlight two potential strategies for development of DAI and mTBI therapies for clinical translation. These data also underscore the importance of investigation into the treatment window post-TBI as secondary injury mechanisms may vary greatly depending on brain region, injury severity, and time post-injury.

## Materials and methods

### Pharmacological compounds

Dynasore (cat. SML1937) and SN-6 (cat. 324410) were purchased from Sigma-Aldrich Corp, St. Louis, MO, USA.

### Device fabrication and assembly

Microfluidic uniaxial strain devices were manufactured using PDMS (Sylgard 184, Dow Corning Corp., Midland, MI, USA) and were comprised of four layers as follows. On top is a PDMS ring used to contain media (ID = 30 mm, OD = 40 mm) made from a 10-mm-thick PDMS sheet. Below is the axon-guiding microchannel layer with microchannels of 50 µm width and 3 µm height separated by 50 µm. Beneath the microchannel layer is the pneumatic deflection layer made of PDMS with a microchannel of 35 mm length, 2 mm width, and 50 µm height. On the bottom is a glass cover slip (#2, 50 mm × 60 mm). Pneumatic deflection and microchannel layers were fabricated using soft lithography replication of SU molds as previously described^30,31,64^.

To generate the microchannel layer mold, SU8-2002 (Microchem, MA, USA) was used to fabricate an array of parallel microchannels (width = 50 μm, height = 3 μm) with a spacing of 50 μm between microchannels. For the pneumatic layer mold, SU8-2025 (Microchem, MA, USA) was used to fabricate a single channel (length = 35 mm, width = 2 mm, and height = 50 μm). The thickness of SU8 and PDMS was characterized with a profilometer (Dektak 3030, Veeco, NY, USA). PDMS curing elastomer and curing agents were added together in 10:1 wt/wt ratio and were mixed and degassed for 15 min to remove any air bubbles. Microchannel and pneumatic channel layers were then immediately fabricated by spinning the PDMS mix on the respective mold with a spin speed of 1000 rpm for 30 s and curing in an oven at 65 °C for 4 h. This resulted in a 79 ± 3-μm-thick patterned PDMS layer on each mold.

Devices were assembled as follows. The PDMS ring was bonded to the PDMS membrane on the microchannel layer mold using oxygen plasma treatment. The membrane was then peeled off the mold and excess membrane outside the ring was trimmed with a scalpel. A laser-cut template with two holes (diameter of 3 mm separated by a 2 mm distance between them) was centered on the membrane inside the ring. Holes were aligned parallel to the length of the microchannels and were generated using a 30-mm punch inside. The entire assembly was then rinsed in 70% ethanol, cleaned with scotch tape, and dried in a 65 °C oven. The cleaned assembly was then bonded to the PDMS membrane on the pneumatic layer mold using oxygen plasma treatment. Microchannels on the microchannel layer were carefully aligned along the diameter of assembly and were perpendicular to the microchannel on the pneumatic channel layer to ensure that the generated pneumatic holes were made on both sides of the pneumatic microchannel. Through holes were punched into three-layer assemblies at each end of the pneumatic microchannel using a 1-mm punch. These pneumatic holes were used to connect tubing for the injection of air pressure to induce uniaxial strain injury. The three-layer assembly was then cleaned again with scotch tape and bonded to cover glass using corona treatment. The manufacturing protocol for both PDMS layers was strictly followed in order to ensure that the material thicknesses and resulting material properties were consistent from batch to batch.

### Organotypic slice culture

All animal studies were performed in accordance with US Department of Health and Human Services Guide for the Care and Use of Laboratory Animals and were approved by the Rutgers University and University of Pennsylvania Institutional Animal Care and Use Committees. The brains of postnatal day 4–8 Sprague Dawley rat pups were removed and placed in ice-cold Gey’s Balanced Salt Solution (Sigma-Aldrich Corp, St. Louis, MO, USA) with 10 mM d-glucose (Sigma-Aldrich) and 3 μM Kyurenic Acid (Sigma-Aldrich). Hippocampi were isolated and sliced into 350-μm-thick slices with the McIllwain Tissue Chopper (Stoelting Co, Wood Dale, IL, USA). Slices from each individual donor animal were kept separated and not pooled. For uniaxial strain injury experiments, organotypic cultures were maintained in PDMS microfluidic devices described above coated with poly-D-lysine (100 μg/ml, Sigma-Aldrich) and laminin (25 μg/ml, Sigma-Aldrich). For oxidative stress experiments, organotypic slices were cultured in 6-well tissue culture plates coated with poly-D-lysine (100 μg/ml). Cultures were maintained in Neurobasal A (supplemented with B27, 2 mM GlutaMAX, and 20 μg/ml gentamycin). Hippocampal organotypic slice cultures were placed on a rocker (~1 revolution/60 s) in a 37 °C and 5% CO_2_ incubator. Media was changed every 48 h and the microfluidic devices were covered with thin sheets of PDMS to prevent evaporation. Uniaxial strain injury experiments were performed on day in vitro (DIV) 3–5 after axonal processes have crossed over the pneumatic channel from one slice chamber to the adjacent chamber. Oxidative stress experiments were performed on DIV 2–4.

### Stretch–strain injury application

To apply uniaxial strain injury, the inlet of the device is connected to a syringe and the outlet is connected to a pressure transducer using plastic tubing with suitable pressure compliance. The syringe piston is attached to a linear actuator (PS01-23 × 80—LinMot USA Inc, Lake Geneva, WI, USA) with controller (E100-MT—LinMot USA Inc) driven by a computer program. A pressure transducer (Honeywell International, NJ, USA) is connected to the ADC converter on an Arduino microcontroller board programmed to send the digital pressure readout to the computer. A custom written visual basic program is used to drive the syringe piston to generate the desired pressure and to monitor the delivery of applied pressure simultaneously. This allows for precise control of injury and improves reproducibility of the desired pressure-based deflection and hence strain with desired strain rate.

### Protein expression analysis

Organotypic cultures and flash-frozen brain tissues were lysed and protein extracts were prepared as follows. Tissue was lysed in RIPA buffer (50 mM Tris–HCl pH 7.4; 150 mM NaCl; 0.5% deoxycholate; 1% NP-40; 1 mM EDTA pH 7.4; 0.1% sodium dodecyl sulfate (SDS)) containing 1 mM phenylmethylsulfonyl fluoride and 1× Protease Inhibitor Cocktail (Roche Diagnostics GmbH, Mannheim, Germany) and 1× PhosSTOP phosphatase inhibitor tablet (Sigma). Tissue was sonicated, placed on rotator for 1 h, and centrifuged at 12,000 × *g* for 15 m at 4 °C. Protein concentrated in supernatant fraction was determined with the Pierce BCA Protein Assay Kit (ThermoFisherScientific, Waltham, MA, USA). Proteins (10–15 μg) were resolved on SDS-polyacrylamide gels and transferred to polyvinylidene difluoride membranes. Membranes were blocked with 5% bovine serum albumin (BSA) in TBST (20 mM Tris pH 7.5; 150 mM NaCl; 0.1% Tween-20) for 1 h. Membranes were probed with the following antibodies in 3% BSA in TBST at 1:1000: anti-NCX1 (ThermoFisherScientific; cat. #MA3-926), anti-DRP1 EPR19274 (abcam, Cambridge, MA, USA; cat. #ab184247), anti-Bax (Cell Signaling Technology, Danvers, MA, USA; cat. #2772 S), anti-cytochrome C D18C7 (Cell Signaling Technology; cat. #11940 S), anti-Bcl-xL 54H6 (Cell Signaling Technology; cat. #2764 S), anti-Bcl-2 (Santa Cruz Biotechnology; cat. sc-7382) anti-α-tubulin (abcam; cat. ab6161), and anti-glyceraldehyde 3-phosphate dehydrogenase (anti-GAPDH; abcam; cat. ab8245). Protein bands were visualized by chemiluminescence with Immobilon Western Chemiluminescent HRP Substrate (Millipore) and the LI-COR Odyssey Fc Imaging system (LI-COR Biosciences, Lincoln, NE, USA). Band intensities were quantified using the LI-COR Image Studio Software and normalized to levels of internal control (α-tubulin or GAPDH).

### Mild TBI

C57Bl/6J mice (male; 10 weeks of age) were anesthetized with isoflurane and placed in a stereotaxic frame on a heating pad. A craniotomy procedure was performed over the left parietotemporal cortex midway between bregma and lambda. For mild CCI injury, a rounded silicone indentor tip impacted the cortex at a velocity of 0.43 m/s and an impact depth of 2 mm. Following application of injury, the craniotomy was sutured and animals were returned to a warm recovery cage.

### Oxidative stress experiments

For oxidative stress experiments, organotypic hippocampal slices were cultured in six-well tissue cultures plates as described above. On day of experiment (DIV 2–4) cultures were treated with vehicle (0.08% dimethyl sulfoxide) or drug compounds for 2 h and subsequently exposed to 2.5 mM H_2_O_2_ (Sigma) for 1 h. All treatment and injury solutions were prepared in complete Neurobasal A. Cultures were washed 3× with phosphate-buffered saline (PBS) between solution changes. Following H_2_O_2_ exposure and PBS washes, respective solutions were placed back into respective culture plate wells. For cell death and oxidative stress experiments, slices were labeled ~2 h after H_2_O_2_ exposure. Slices were incubated with 3 μM PI for 30 min and CellROX™ Green Reagent (ThermoFisherScientific) for 1 h and washed 3× with PBS prior to measurements. Phase-contrast and fluorescence micrographs were taken under a ×4 objective using the EVOS FL Microscope (ThermoFisherScientific; serial #: I2414-155G-405). Light and exposure settings were kept constant throughout experiments. Relative fluorescence intensity was measured using ImageJ (NIH) in respective regions of the hippocampus.

### Axonal and mitochondrial morphology analysis

Axonal phase-contrast and mitochondrial fluorescence micrographs were taken using the EVOS FL Microscope (ThermoFisherScientific; serial #: I2414-155G-405) under a ×20 objective. Axonal varicosity area was measured using ImageJ. Random ROIs (100 μm × 50 μm) were selected and axonal phase-contrast images were manually traced and measured in ImageJ. Varicosity number was manually counted along 60–80 μm of each axonal process with the experimenter blinded to the condition. Mitochondrial morphology was analyzed using ImageJ. Mitochondria were labeled with Mitotracker Green FM (ThermoFisherScientific) as per the manufacturer’s instructions. Random ROIs (~10 μm × 20 μm) were selected, mitochondrial signal was thresholded, and morphology was measured using a custom ImageJ macro. The same threshold percentage was used across ROIs.

### Immunohistochemistry and microscopy

Slice cultures were fixed with 4% paraformaldehyde overnight at 4 °C and incubated in blocking buffer (10% normal goat serum in 0.1% Triton-X in PBS) for 1 h. Cultures were left in primary antibody (1:200 anti-tau, abcam; cat. #ab75714), 1:500 anti-MAP2 (BD Pharmingen, San Diego, CA, USA; cat. #556320), 1:200 anti-DRP1 EPR19274 (abcam; cat. #ab184247), 1:200 anti-NCX1 EPR12739 (abcam; cat. #ab177952), and 1:200 anti-TOMM20 (abcam; cat. #ab56783) overnight at 4 °C, washed 3× with PBS, and incubated with respective secondary antibody conjugated to Alexa Fluor 488 or 647 (1:500; ThermoFisherScientific). Fluorescence micrographs were taken under a ×20 objective using the EVOS FL Microscope (ThermoFisherScientific). The following filter cubes were used for this study: EVOS™ Light Cube, GFP cat. AMEP4651; EVOS™ Light Cube, RFP cat. AMEP4652; EVOS™ Light Cube, Cy™5 cat. AMEP4656. The Look Up Table (LUT) applied to display CellROX Green fluorescence intensity is linear and found in ImageJ (16-color LUT). Colocalization of TOMM20 and Drp1 immunostaining was determined using the Coloc 2 plugin (https://imagej.net/Coloc_2) in ImageJ.

### Statistics

Graphpad Prism 7 software was used to perform all statistical analyses.

## Supplementary information

Supplementary Fig. S1

Supplementary Fig. S2
