# Supplementary material for: Dynamin and reverse-mode sodium calcium exchanger blockade confers neuroprotection from diffuse axonal injury
Source: Cell Death Dis. 2019 Sep 27;10(10):727. doi: 10.1038/s41419-019-1908-3 (PMC6765020; doi:10.1038/s41419-019-1908-3)
Supplement: Supplementary file 1 — Supplementary Fig. S1 [file 41419_2019_1908_MOESM1_ESM.pdf]

Supplementary Figure S1

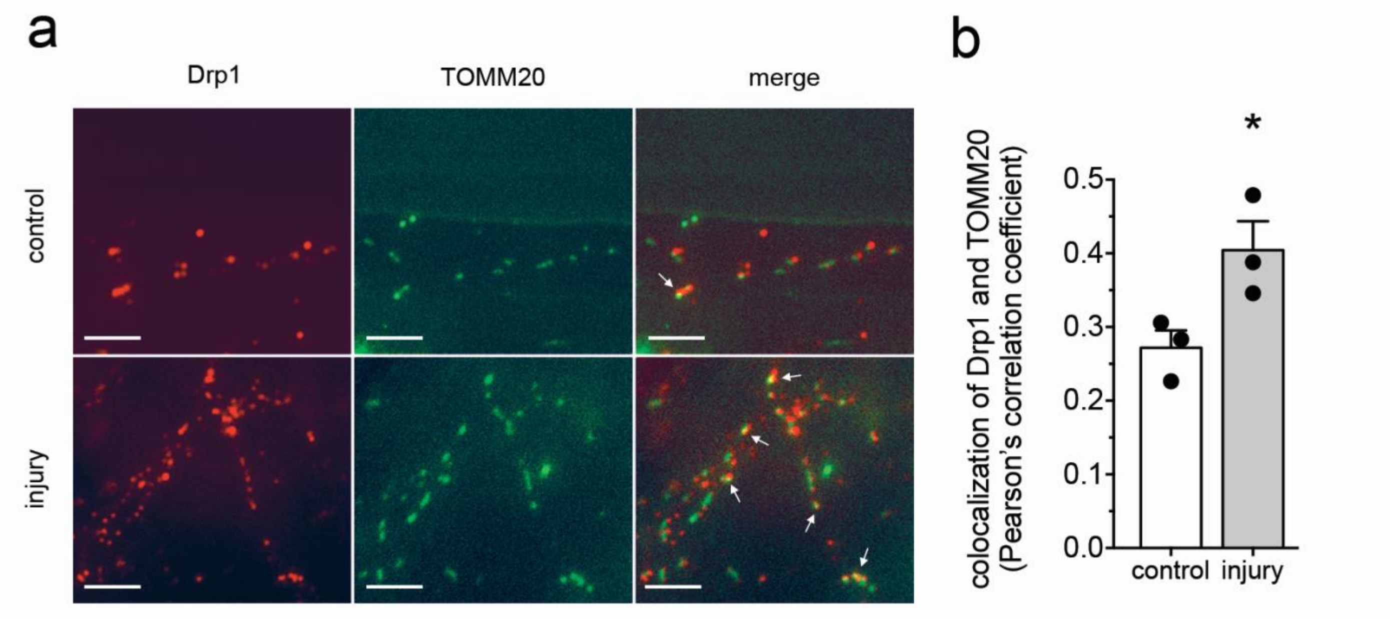


**Supplementary Fig. S1** Colocalization analysis of Drp1 and TOMM20 immunostaining in control and injury conditions. Axons protruding from hippocampal organotypic slices cultured in microfluidic devices were injured with application of 15% strain and fixed 5min post-injury. **a.** Representative fluorescence micrographs displaying Drp1 and TOMM20 immunostaining in axonal processes in control and injury conditions. White arrows indicate regions of colocalization. **b.** Analysis shows increased colocalization of Drp1 and TOMM20 following axonal stretch injury. Results obtained from three microfluidic device cultures per condition (separate donor animals used for each device), n = 3. *$p < 0.05$ as determined by Student's *t*-test. Error bars are s.e.m. Scale bars, 5µm.
