# Supplementary material for: Dynamin and reverse-mode sodium calcium exchanger blockade confers neuroprotection from diffuse axonal injury
Source: Cell Death Dis. 2019 Sep 27;10(10):727. doi: 10.1038/s41419-019-1908-3 (PMC6765020; doi:10.1038/s41419-019-1908-3)
Supplement: Supplementary file 2 — Supplementary Fig. S2 [file 41419_2019_1908_MOESM2_ESM.pdf]

Supplementary Figure S2

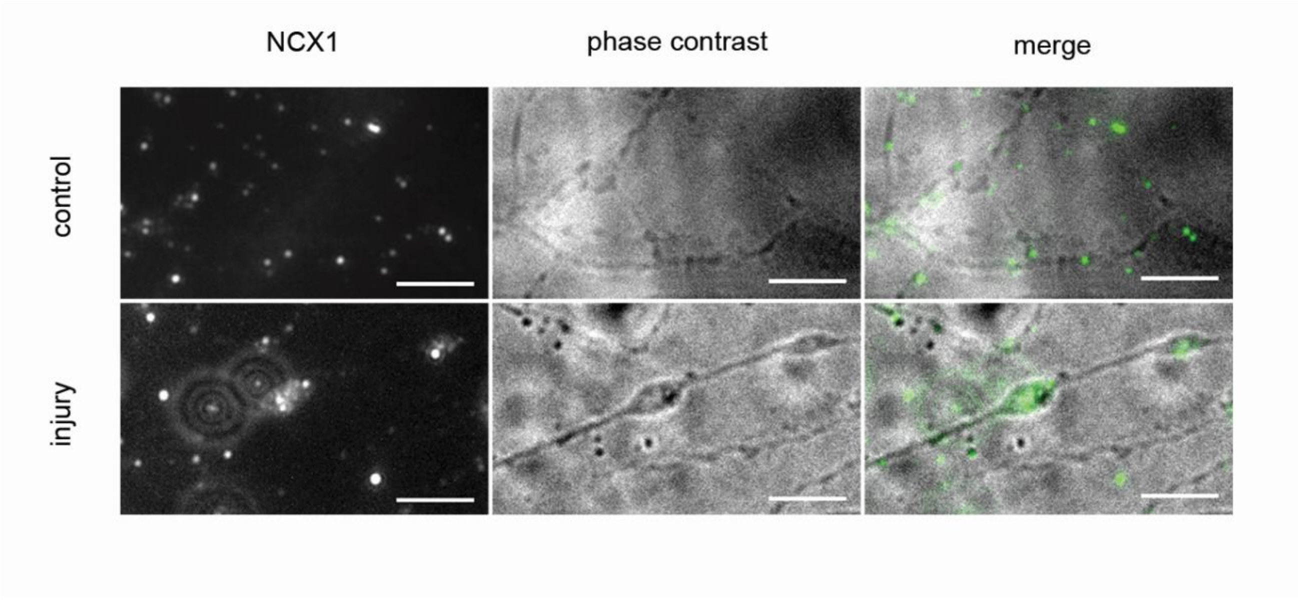

Supplementary Fig. S2 NCX1 immunostaining in control and injury conditions. Axons protruding from hippocampal organotypic slices cultured in microfluidic devices were injured with application of 15% stretch and fixed 5min post-injury. Representative fluorescence micrographs showing NCX1 immunostaining in axonal processes in control and injury conditions. Results obtained from three microfluidic device cultures per condition (separate donor animals used for each device), n = 3. Scale bars, 20µm.
